# Synacthen Stimulation Test Following Unilateral Adrenalectomy Needs to Be Interpreted With Caution

**DOI:** 10.3389/fendo.2021.654600

**Published:** 2021-05-11

**Authors:** Shamaila Zaman, Raya Almazrouei, Amir H. Sam, Aimee N. DiMarco, Jeannie F. Todd, Fausto F. Palazzo, Tricia Tan, Waljit S. Dhillo, Karim Meeran, Florian Wernig

**Affiliations:** ^1^ Department of Endocrinology, Hammersmith Hospital, Imperial College Healthcare NHS Trust, London, United Kingdom; ^2^ Endocrine Division, Tawam Hospital, Al Ain, United Arab Emirates; ^3^ Division of Diabetes, Endocrinology and Metabolism, Imperial College London, London, United Kingdom; ^4^ Department of Endocrine and Thyroid Surgery, Hammersmith Hospital, Imperial College Healthcare NHS Trust, London, United Kingdom

**Keywords:** adrenalectomy, synacthen stimulation test, adrenal insufficiency, over-night dexamethasone suppression test, autonomous cortisol secretion

## Abstract

**Background:**

Cortisol levels in response to stress are highly variable. Baseline and stimulated cortisol levels are commonly used to determine adrenal function following unilateral adrenalectomy. We report the results of synacthen stimulation testing following unilateral adrenalectomy in a tertiary referral center.

**Methods:**

Data were collected retrospectively for 36 patients who underwent synacthen stimulation testing one day post unilateral adrenalectomy. None of the patients had clinical signs of hypercortisolism preoperatively. No patient received pre- or intraoperative steroids. Patients with overt Cushing’s syndrome were excluded.

**Results:**

The median age was 58 (31-79) years. Preoperatively, 16 (44%) patients had a diagnosis of pheochromocytoma, 12 (33%) patients had primary aldosteronism and 8 (22%) patients had non-functioning adenomas with indeterminate/atypical imaging characteristics necessitating surgery. Preoperative overnight dexamethasone suppression test results revealed that 6 of 29 patients failed to suppress cortisol to <50 nmol/L. Twenty (56%) patients achieved a stimulated cortisol ≥450 nmol/L at 30 minutes and 28 (78%) at 60 minutes. None of the patients developed clinical adrenal insufficiency necessitating steroid replacement.

**Conclusions:**

Synacthen stimulation testing following unilateral adrenalectomy using standard stimulated cortisol cut-off values would wrongly label many patients adrenally insufficient and may lead to inappropriate prescriptions of steroids to patients who do not need them.

## Introduction

Adrenal insufficiency (AI) is caused by failure of the adrenal cortex to produce adequate amounts of corticosteroids and is associated with increased morbidity and mortality (1). Unilateral adrenalectomy can be a rare cause of adrenal insufficiency. Patients with unilateral adrenal cortisol-producing lesions (adrenal Cushing’s syndrome) may develop adrenal insufficiency after adrenalectomy due to contralateral adrenal suppression (2). Similarly, patients with autonomous cortisol secretion (subclinical Cushing’s syndrome) are considered to be at risk of adrenal insufficiency following adrenalectomy (2).

The synacthen stimulation test (SST) remains the most widely used test in investigating AI and has been validated against the ‘gold standard’, the insulin tolerance test (ITT) in secondary (central) AI (1, 3). A 30-minute stimulated cortisol level in response to intramuscular or intravenous injection of 250 micrograms of synacthen has been used as a criterion to define adrenal insufficiency (3). For various reasons, SSTs alone may not be applicable in the immediate post unilateral adrenalectomy setting to reliably diagnose adrenal insufficiency (4, 5).

We performed a retrospective analysis of SSTs performed on day one after laparoscopic unilateral adrenalectomy for non-Cushing’s syndrome adrenal lesions to determine whether synacthen-stimulated cortisol can be used to detect clinically significant post-operative adrenal insufficiency necessitating steroid replacement therapy.

## Materials and Methods

### Patients Cohort

Data were collected retrospectively for patients who underwent post-operative SST after unilateral adrenalectomy for non-cortisol secreting lesions in a tertiary care center between April 2016 to March 2019. SST was performed routinely on all patients on day one post-adrenalectomy. The majority of procedures were carried out laparoscopically except four patients who underwent open adrenalectomy (with three patients converted from minimally invasive surgery to an open procedure). The median length of stay was two days. The study was approved by the Imperial College Healthcare NHS Trust governance team who confirmed that as we are reporting on routinely collected non-identifiable clinical audit data, no approval from a research ethics committee was additionally required under the UK policy framework for Health and Social Care.

### Post-Operative SST

Post-operative SST was performed on day one postoperatively. Cortisol was measured between 8 and 9 am followed by intramuscular injection of 250 micrograms of tetracosactide. Cortisol was measured at 30 and 60 minutes following the injection (6).

### Cortisol Measurement

Serum cortisol levels were measured using the Abbott Architect i‐2000 immunoassay analyser (Abbott Diagnostics, Maidenhead, UK). The precision was ≤10% total coefficient of variation (CV) for serum samples ≥83 to ≤965 nmol/L, the limit of detection was ≤22 nmol/L. The cross-reactivity of cortisone in this assay is minimal (2.7% at 1000 µg/dl). A stimulated serum cortisol of ≥450 nmol/L at 30 minutes was regarded as normal which is the local cut-off derived from reference comparison work with older assays. In addition, a lower stimulated cortisol cut-off of 375 nmol/L was applied because peak stress cortisol response to major surgery in euadrenal patients had previously been shown to range from 375 to 1452 nmol/L (7).

One mg over-night dexamethasone suppression tests (ONDST) were performed (8) as part of functional status evaluation of adrenal incidentalomas or to exclude cortisol co-secretion in aldosterone-secreting adenomas. Results were collected whenever available from electronic health care records or referral letters. None of our patients were on oral contraceptive medications, hormone replacement therapy or steroids pre-operatively. As stated above, only patients with non-cortisol secreting adrenal lesions were included. Patients undergoing bilateral adrenalectomy, patients with Cushing’s syndrome, patients with adrenal cortical carcinoma and patients who received intraoperative steroids were excluded.

### Statistical Analysis

Proportional outcomes were presented as numbers and percentages. Continuous values were expressed as mean with standard deviation (SD) or median with range if normally or non-normally distributed respectively. We performed Chi-Square and Fisher’s Exact tests for categorical variables, t tests for normally distributed variables and Mann-Whitney U test and Kruskal-Wallis test for non-normally distributed variables. A two-tailed probability value of <5% was considered statistically significant. Statistical analysis was performed using statistical software (Stata13, Stata corp LP, Texas).

## Results

The study included 36 patients with a median age of 58 years and a female to male ratio of 1.8. Sixteen (44%) patients had a diagnosis of phaeochromocytoma, 12 (33%) patients had primary aldosteronism and 8 (22%) patients had non-functioning lesions with indeterminate/atypical imaging characteristics necessitating surgery (**Table 1**).

**Table 1:** Baseline characteristics of patients who underwent short synacthen testing post unilateral adrenalectomy.

Number	36
Median Age (5-95% range)	58 (31-79)
Male gender (N, %)	23 (64%)
• Preoperative Diagnosis (N, %):	
• Pheochromocytoma	16 (44%)
• Primary aldosteronism	12 (33%)
Non-functioning lesions	8 (22%)
Median lesion size in cm (5-95% range) N=30*	3.5 (1-7)
• Pheochromocytoma (N=16)	4.25 (2-8)
• Primary aldosteronism (N=6)	1.35 (0.8-2)
• Non-functioning lesions (N=8)	4.75 (2-7)
Morning cortisol post ONDST (N=29)	
‐ ≤50 nmol/L	23 (79%)
‐ 51-138 nmol/L	5 (17%)
‐ >138 nmol/L	1 (3%)

*Lesion size based on histology. Six patients who underwent surgery for primary aldosteronsim had adrenal hyperplasia on histology.

*Histological diagnosis of 8 non-functioning lesions include adenomas (3), cyst (1), leiyomyosarcoma (2), myolipoma (1), granulomatous lesion (1).

Overnight dexamethasone suppression test results were available for 29 patients, 15 for patients with a diagnosis of phaeochromocytoma, 10 for patients with primary hyperaldosteronism and 4 for patients with non-functioning lesions. Following dexamethasone administration, 23 (79%) patients had cortisol levels of ≤50 nmol/L (14 phaeochromocytoma patients, 7 patients with primary aldosteronism and 2 patients with non-functioning lesions), 5 patients had cortisol levels between 51-138 nmol/L (1 patient with pheochromocytoma, 2 patients with primary aldosteronism and 2 patients with non-functioning lesions) and only one patient had a cortisol level of >138 nmol/L with a primary diagnosis of primary aldosteronism (**Table 1**).

**Figure 1:**
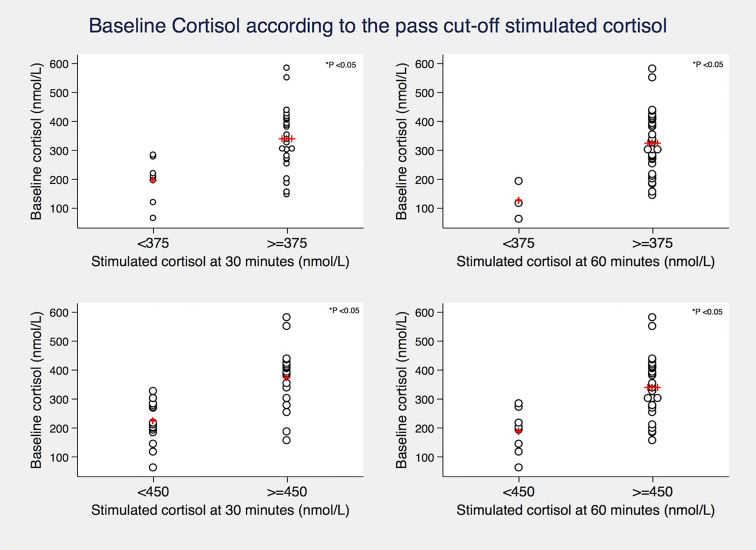
Relationship between baseline cortisol levels and different synacthen-stimulated cortisol cut-offs (375 and 450 nmol/L) at 30 and 60 minutes. Red crosses represent mean values. *P* value derived from Two-sample T-test.

**Figure 2:**
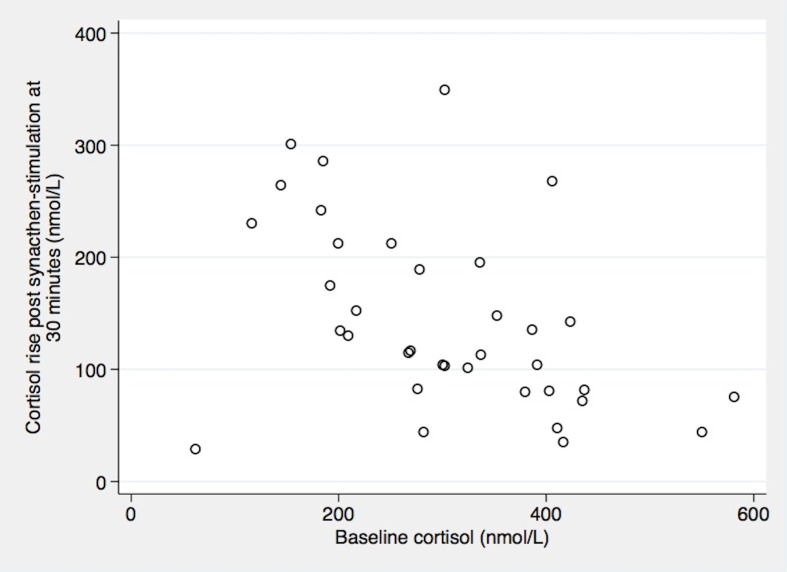
Relation between baseline cortisol and cortisol rise post synacthen-stimulation at 30 minutes. Correlation Coefficient Spearman’s rho= -0.52 (*P* 0.0012).

**Figure 3:**
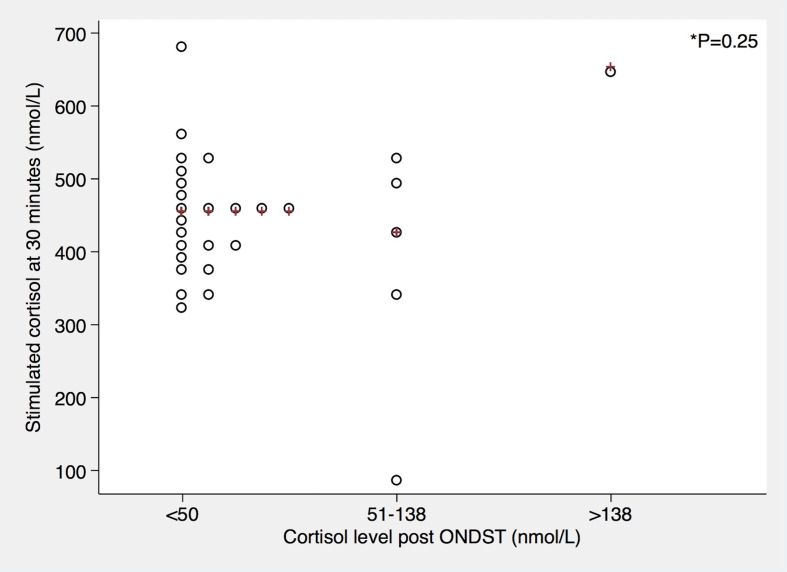
Relationship between dexamethasone-suppressed cortisol levels and 30 minute synacthen-stimulated cortisol levels. Red crosses represent median values. *P*-value was derived from Kruskal-Wallis test.

Using a cut-off for stimulated cortisol of 450 nmol/L at 30 minutes, 20/36 (56%) of patients passed the SST. Using a lower cut-off of 375 nmol/L, 28/36 (78%) patients would have passed at 30 minutes. When considering 60 minute values of stimulated cortisol, an 28/36 (78%) and 33/36 (92%) patients would pass the test when using a 450 nmol/L and 375 nmol/L cut-off respectively (**Table 2**). Mean baseline cortisol levels were significantly higher in those who passed the cut-off than in those who did not (*p value <0.05*) (**Figure 1**). However, the synacthen-stimulated cortisol rise at 30 minutes was greater in those patients with lower baseline cortisol values (**Figure 2**). Underlying diagnosis or ONDST results did not result in significantly different peak cortisol levels following synacthen stimulation (**Table 2** and **Figure 3**). All patients were followed up clinically and those patients who were followed up in different centres were contacted by the research team following surgery. Importantly, none of the patients required steroid replacement or developed features of adrenal insufficiency during a minimum of 12 month follow-up period post-surgery.

**Table 2:** Distribution of ONDST, baseline cortisol and histology based on different peak of cortisol cut-offs.

	30 minutes Peak Cortisol ≥450 nmol/L N= 20	30 minutes Peak Cortisol <450 nmol/L N= 16	P Value
**Baseline Cortisol in SST** (Mean +SD)	371 ± 104	222 ± 73	0.00001^a^
**ONDST:**			0.801^b^
<50	13	10	
51-138	2	3	
>138	1	0	
**Histology:**			
Pheochromocytoma	6	10	0.151^b^
Primary Aldosteronism	9	3	
Non-Functioning Lesions	5	3	
	**60 minutes Peak Cortisol ≥450 nmol/L N=28**	**60 minutes Peak Cortisol <450 nmol/L N=8**	
**Baseline Cortisol in SST** (Mean +SD)	339 ± 105	186 ± 74	0.0005^a^
**ONDST:**			
<50	18	5	0.677^b^
51-138	3	2	
>138	1	0	
**Histology:**			
Pheochromocytoma	12	4	0.775^b^
Primary Aldosteronism	9	3	
Non-Functioning Lesions	7	1	
	**30 minutes Peak Cortisol ≥375 nmol/L N=28**	**30 minutes Peak Cortisol <375 nmol/L N=8**	
**Baseline Cortisol in SST** (Mean +SD)	336 ± 109	195 ± 74	0.0016^a^
**ONDST:**			0.433^b^
<50	19	4	
51-138	3	2	
>138	1	0	
**Histology:**			0.886^b^
Pheochromocytoma	12	4	
Primary Aldosteronism	10	2	
Non-Functioning Lesions	6	2	
	**60 minutes Peak Cortisol ≥375 nmol/L N=33**	**60 minutes Peak Cortisol <375 nmol/L N=3**	
**Baseline Cortisol in SST** (Mean +SD)	321 ± 107	124 ± 65	0.004^a^
**ONDST:**			0.515^b^
<50	21	2	
51-138	4	1	
>138	1	0	
**Histology:**			0.435^b^
Pheochromocytoma	14	2	
Primary Aldosteronism	12	0	
Non-Functioning Lesions	7	1	

a Significant difference (Two sample T-Test, alpha=0.05).

b Non significant difference (Fisher Exact Test, alpha=0.05).

## Discussion

Honda et al. observed a compensatory rise in plasma ACTH levels in patients undergoing unilateral adrenalectomies for primary aldosteronism but did not find any significant difference in pre-and post-operative basal cortisol levels. Stimulated peak cortisol levels remained lower following adrenalectomy, but none of the patients developed clinically relevant adrenal insufficiency. Patients with subclinical Cushing’s syndrome had been excluded in this study (4). Similarly, a review of 331 adrenalectomies over a period of 12 years showed that no patient undergoing unilateral adrenalectomy for non-cortisol producing lesions required steroid replacement post-operatively. Those who did require steroid replacement, had a diagnosis of Cushing’s syndrome or subclinical Cushing’s syndrome (9). Our own study excluded patients with overt Cushing’s syndrome. Based on ONDST results, 6 patients had possible or autonomous cortisol secretion (without clinical features of hypercortisolism). SSTs were carried out on day 1 following adrenalectomy as most patients would be discharged on day 1 or day 2 following laparoscopic surgery. None of our patients were given post-operative steroid replacement despite the fact that 44% of patients did not reach a 30 minute cortisol peak of more than 450 nmol/L. Tang et al. report a single centre experience of 22 patients with aldosterone and cortisol co-secreting adenomas and 392 aldosterone-secreting adenomas. All patients were given peri-operative steroids which were weaned off over the course of one week following surgery. No episode of adrenal crisis or adrenal insufficiency was observed in this study (10). One would expect patients with autonomous cortisol secretion to be at higher risk of postoperative adrenal insufficiency due to contralateral adrenal suppression (2, 11). Autonomous cortisol secretion in the absence of overt clinical manifestations of Cushing syndrome can be found in up to 20-25% of adrenal tumors (12). Current guidelines suggest that a serum cortisol level of less than 50 nmol/L post 1mg of dexamethasone excludes cortisol hyper-secretion in patients with adrenal incidentalomas. For patients without overt Cushing’s syndrome and a serum cortisol post dexamethasone between 51 and 138 nmol/L, the term ‘possible autonomous cortisol secretion’ is proposed and those with serum cortisol more than 138 nmol/L, the term ‘autonomous cortisol secretion’ is used in the absence of clinical manifestations of overt Cushing’s syndrome (3). When using ONDST as the sole criteria it is important to remember that the diagnostic accuracy of ONDST can be affected by variable absorption and metabolism of dexamethasone as well as by drug interference (12). In our patient cohort, we did not find a significant difference in post-operative stimulated peak cortisol levels at 30 minutes based on the pre-operative ONDST categories, but our sample size was too small to draw firm conclusions and only one patient had a post-ONDST cortisol of >13nmol/L (**Figure 3**). Similar to the results reported by others (10), none of our patients developed clinical adrenal insufficiency until at least 12 months post-operatively. One of our patients was recommended steroid replacement as he only achieved a post-operative synacthen stimulated peak cortisol level of 91 nmol/L. This patient’s dexamethasone-suppressed cortisol was 78 nmol/L before surgery. Against medical advice, the patient elected not to take steroids and remained clinically well. A repeat SST nine months later showed a 30 minute cortisol level of 480 nmol/L. Whilst this particular patient might have temporarily required steroid replacement in the event of stress, most patients who failed the SST following unilateral adrenalectomy using established cut-off criteria do not need steroid replacement.

In addition, after the unilateral adrenalectomy, the remaining adrenal gland has adequate capacity to make up for the resected gland over time.4 As contralateral adrenal hypertrophy takes time. SST test done in the immediate postoperative period would be premature, and those who fail the test may require observation only.

It therefore appears that synacthen-stimulated cortisol using established cortisol cut-offs cannot accurately define patients at risk of adrenal insufficiency following unilateral adrenalectomy for non-cortisol secreting adrenal lesions. A recent prospective study of 100 patients with primary aldosteronism post-adrenalectomy found that 27% of patients did not reach a synacthen-stimulated cortisol peak of 469 nmol/L (classified having adrenal insufficiency) with 48% of those patients not reaching a peak of 372 nmol/L (classified having severe adrenal insufficiency). The SST was carried out on day 4 or 5 after surgery. The study included patients with autonomous cortisol secretion (5). Honda et al. have shown that the cortisol-secreting capacity is reduced following unilateral adrenalectomy, but this reduction does not lead to any significant clinical consequences in most cases. Using synacthen-stimulated cortisol as the only marker to determine adrenal insufficiency would lead to overuse of steroid replacement and in some cases result in increased morbidity associated with increased steroid replacement doses (13–15).

Synacthen-stimulated cortisol at 30-minute has been validated against the ‘gold standard’, the insulin tolerance test (1, 3). However, most studies either included healthy subjects or patient with diagnosed or suspected AI (6, 16, 17). Cut-offs to define adrenal insufficiency derived from those studies may not be applicable to an immediate post-adrenalectomy setting. We have demonstrated previously that the cortisol response in the immediate post-operative period in euadrenal patients undergoing different types of surgery can be highly variable. Those who underwent major surgery had serum cortisol levels ranging from 375 nmol/L to 1452 nmol/L with a median serum cortisol of 680 nmol/L. Opioid use, lower cortisol binding globulins and albumin in the post-operative period also affect the results (18, 19). Cortisol binding globulin is significantly lower following surgery as shown previously (20), and the free cortisol index may therefore be much more reliably reflect true adrenal reserve post unilateral adrenalectomy. In addition, when defining cut-off levels, it is important to remember that cortisol levels are assay specific (21). Therefore, a lower threshold might more accurately identify patients at risk of clinically relevant transient adrenal insufficiency. Undoubtedly, there is a ‘grey zone’ where patients are unlikely going to suffer an adrenal crisis, but could potentially be harmed by unnecessary use of steroid replacement therapy. Definitions of adrenal insufficiency used in other studies were based on cut-off values only and not on clinical symptoms (2, 5).

Our local protocol included ONDST for all patients with adrenal lesions including those with phaeochromocytomas as ectopic ACTH production is known to be a rare cause of Cushing’s syndrome in patients with phaeochromocytomas. Such a case reported by us recently describes a patient with phaeochromocytoma who developed severe Cushing’s syndrome, which was only discovered during his elective admission for adrenalectomy (22). In addition, there is a recent study published which showed higher cortisol concentrations in patients with phaeochromocytomas than in patients with primary hypertension (23).

Strengths of our study were that patients undergoing adrenalectomy for different types of non-cortisol secreting adrenal lesions were included. All adrenalectomies were carried out by a single surgeon in a tertiary referral centre. Serum cortisol was assessed using the same cortisol assay.

Limitations to our study include its retrospective nature and the limited sample size. Not all patients had available pre-operative ONDST results. Synacthen stimulation testing was carried out on day one following surgery as most patients would be discharged the day after surgery. Patients did not routinely undergo follow-up stimulated synacthen testing, but were followed up clinically for a period of at least 12 months. We did not have a control group of euadrenal patients undergoing non-adrenal abdominal surgery.

## Conclusion

The routine use of conventional synacthen-stimulated cortisol cut-off levels in the immediate post-operative setting would over-diagnose adrenal insufficiency following unilateral adrenalectomy and lead to unnecessary steroid replacement therapy. SST should be reserved for patients at risk of post-operative adrenal insufficiency, such as those with pre-operative autonomous cortisol secretion. Lower stimulated cortisol cut-offs and use of free cortisol index may more accurately identify patients at risk of adrenal insufficiency.

## Data Availability Statement

The original contributions presented in the study are included in the article/supplementary material. Further inquiries can be directed to the corresponding authors.

## Ethics Statement

The studies involving human participants were reviewed and approved by Clinical Audit Committee. Imperial College Healthcare NHS Trust. Written informed consent for participation was not required for this study in accordance with the national legislation and the institutional requirements.

## Author Contributions

SZ and RA: first author. RA, AS, AM, JT, FP, TT, WD, and KM: Senior authorship. FW: Senior and Last authorship. All authors contributed to the article and approved the submitted version.

## Funding

This research did not receive any specific grant from any funding agency in the public, commercial or not-for-profit sector.

## Conflict of Interest

The authors declare that the research was conducted in the absence of any commercial or financial relationships that could be construed as a potential conflict of interest.
